# *Staphylococcus aureus* Bacteremia from Diffuse Muscular Infection Following Acupuncture Visualized by ^18^F-FDG PET/CT and MRI

**DOI:** 10.3390/diagnostics7040059

**Published:** 2017-11-30

**Authors:** Andreas Knudsen, Carsten Thomsen, Lothar Wiese

**Affiliations:** 1 Department of Internal Medicine, Zealand University Hospital, 4000 Roskilde, Denmark; low@regionsjaelland.dk; 2 Department of Radiology, Zealand University Hospital, 4000 Roskilde, Denmark; cert@regionsjaelland.dk

**Keywords:** *Staphylococcus aureus* bacteremia, 18-F-FDG PET/CT, MRI, acupuncture

## Abstract

We describe the clinical course of a 60-year old male admitted with *Staphylococcus aureus* bacteremia and back-pain. The patient was suspected of having spondylitis and treated as such with antibiotics; however, both fluorine-18 fluoro-2-deoxy-d-glucose (18F-FDG) positron-emission tomography and magnetic resonance imaging (MRI) with iv contrast showed significant inflammation of muscles and subcutaneous soft tissue in relation to the patients back and left shoulder, but no signs of the working diagnosis of spondylitis. The unusual location of the infection was not explained until a few days prior to being discharged when the patient reported visits to a local physiotherapist where he would have acupuncture performed for non-specific back pain. His last acupunctural procedure had been performed 6 days prior to admission. This case is, to our knowledge, the first to show muscular inflammation on both 18-F-FDG PET/CT and MRI following acupuncture due to *S. aureus*. This case highlights the need for clinicians to search for alternative explanations when imaging does not support the diagnosis.

**Figure 1:**
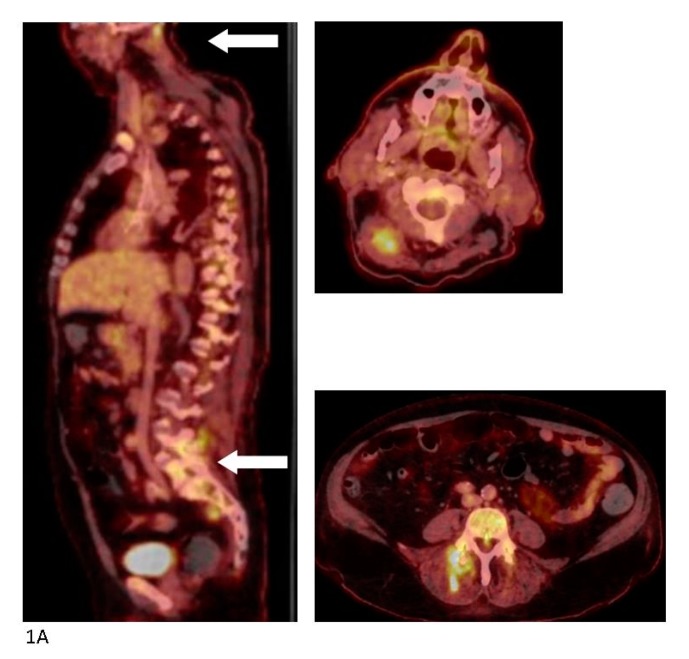

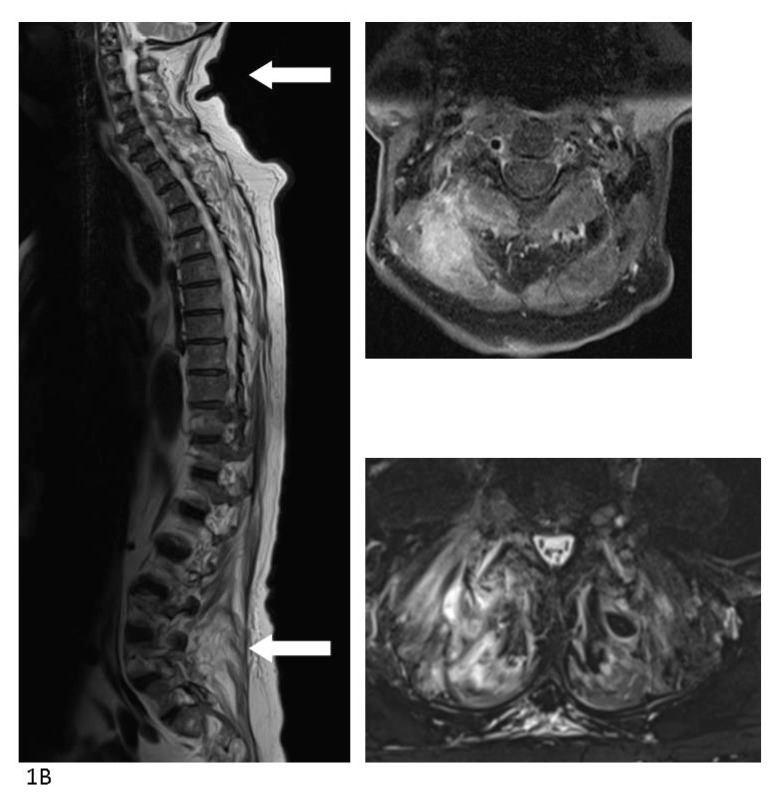
A 64-year old man was admitted to a local hospital with sudden onset of fever, difficulty breathing, and chronic chest and back pain. The patient’s prior medical history included stroke with minor sequelae, well-regulated hypertension, moderate alcohol abuse, and sleep apnea. He was on dual anti-hypertensive medication, muscle relaxants, and blood-thinner. The patient lived alone receiving help for cleaning and shopping. The patient was admitted septic and not fully oriented with a temperature of 39.6 °C, blood pressure of 74/50 mmHg, 95% saturation on 2L O_2_. Following regional guidelines, treatment with piperacillin/tazobactam was initiated. A computerized tomography (CT) with intravenous (IV) contrast performed on the suspicion of pyelonephritis/abdominal focus revealed no pathological findings. Endocarditis was ruled out by trans-esophageal echocardiography. His blood cultures grew methicillin susceptible *Staphylococcus aureus* (MSSA) and dicloxacillin and later rifampicin were added to the treatment. No longer septic, the patient was moved to the section for infectious diseases at the regional university hospital. The patient’s main complaint was diffuse pain in the entire back, resulting in the working diagnosis of bacterial (MSSA) spondylitis. A fluorine-18 fluoro-2-deoxy-d-glucose (^18^F-FDG) positron-emission tomography (PET) combined with a diagnostic CT scan with iv contrast was performed. High FDG uptake was found in the left shoulder and several muscular loci in the musculi semispinalis capitis, piriformis, and gluteus maximus dexteres as well as the lumbar part of the erector spinae, but no signs of spondylitis. FDG uptake in the transverse colon prompted a high suspicion of malignancy. (**A**) The PET/CT scan was supplemented a few days later by a magnetic resonance imaging (MRI) with iv contrast which showed significant inflammation of the muscles—most intensely in the neck and lumbar region and again no signs of spondylitis. (**B**) A slow recovery on physical therapy and iv antibiotics was interrupted by sudden deterioration possibly due to aspiration pneumonia with admission to the intensive care unit for several days. Colonoscopy with biopsy ruled out colonic cancer but found a high-grade neoplasia of the colon and the surgeons made plans for further work-up. Slow but steady recovery continued and a second MRI with iv contrast four weeks after the initial showed less inflammation of all muscular foci. At that point, during rounds, the patient revealed that he regularly received acupuncture of the entire back and shoulder due to muscular tensions and that he had done so for several years. The last session of acupuncture was performed six days prior to the initial admission at the local hospital. He described that the acupuncture was performed at the local physiotherapist, and that he during the last session had placed 24 needles from the neck down to the buttocks. No anti-septics were used during the session. The patient was discharged after eight weeks of iv antibiotic treatment with another eight weeks of oral antibiotics for ambulant follow-up with the department for infectious diseases and municipal physiotherapy. Both local and systemic infections following acupuncture have been reported previously with different organ manifestations and the causative microorganism is most commonly *Staphylococcus aureus* [1,2,3]. With the widespread use of acupuncture for very different medical conditions in settings with no supervision or hygiene regulation, the use of this procedure should be investigated in cases with systemic infection or in cases with several infectious loci, especially when the imaging does not match the initial diagnosis.
